# Supplementary material for: The Effects of Multidisciplinary Team Meetings on Clinical Practice for Colorectal, Lung, Prostate and Breast Cancer: A Systematic Review
Source: Cancers (Basel). 2021 Aug 18;13(16):4159. doi: 10.3390/cancers13164159 (PMC8394238; doi:10.3390/cancers13164159)
Supplement: Supplementary file 1 [file cancers-13-04159-s001.zip › Supplemental tables and figures - Systematic review.pdf]

cancers

MDPI

Systematic Review

The Effects of Multidisciplinary Team Meetings on Clinical Practice for Colorectal, Lung, Prostate and Breast Cancer: A Systematic Review

Lejla Kočo [1,*,†], Harm H. A. Weekenstroo [1,*,†], Doenja M. J. Lambregts [2], J. P. Michiel Sedelaar [3], Mathias Prokop [1], Jurgen J. Fütterer [1] and Ritse M. Mann [1,2]

Table S1. Database search string.

Sub elements	Mesh or Search term
**Colorectal cancer**	*Mesh*: Colorectal Neoplasms (pubmed), [Colorectal Neoplasms] explode all trees (Cochrane)<br><br>*Search terms*: Colorectal AND cancer/cancers/neoplasm/neoplasms/carcinoma/ carcinomas/tumor/tumors/tumour/tumours/malignancy/malignancies/adenocarcinoma/ adenocarcinomas
**Lung cancer**	*Mesh*: "Lung Neoplasms" (pubmed) , [Lung Neoplasms] explode all trees (Cochrane)<br><br>*Search* terms: Pulmonary/Lung AND cancer/cancers/neoplasm/neoplasms/carcinoma/ carcinomas/tumor/tumors/tumour/tumours/malignancy/malignancies/adenocarcinoma/ adenocarcinomas
**Prostate cancer**	*Mesh*: "Prostatic Neoplasms" (pubmed) , [Prostatic Neoplasms] explode all trees (Cochrane)<br><br>*Search* terms: Prostate/Prostatic AND cancer/cancers/neoplasm/neoplasms/carcinoma/ carcinomas/tumor/tumors/tumour/tumours/malignancy/malignancies/adenocarcinoma/ adenocarcinomas
**Breast cancer**	*Mesh*: "Breast Neoplasms" (pubmed) , [Breast Neoplasms] explode all trees (Cochrane)<br><br>*Search terms*: Breast/Mammary AND cancer/cancers/neoplasm/neoplasms/carcinoma/ carcinomas/tumor/tumors/tumour/tumours/malignancy/malignancies/adenocarcinoma/ adenocarcinomas
**MDTM**	*Mesh*: "Patient Care Team" (pubmed), [Patient Care Team] this term only (Cochrane)<br><br>*Search terms*: Interdisciplinary approach, Interdisciplinary board, Interdisciplinary cancer conference, Interdisciplinary care, Interdisciplinary coordinated care, Interdisciplinary group, Interdisciplinary health care, Interdisciplinary healthcare, Interdisciplinary intervention, Interdisciplinary model, Interdisciplinary patient management, Interdisciplinary team, Interdisciplinary telecare, Interdisciplinary treatment, Interdisciplinary Health Team, MDT, MDTs, Multidisciplinary approach, Multidisciplinary board, Multidisciplinary cancer conference, Multidisciplinary care, Multidisciplinary coordinated care, Multidisciplinary group, Multidisciplinary health care, Multidisciplinary Health Team, Multidisciplinary healthcare, Multidisciplinary intervention, Multidisciplinary model, Multidisciplinary patient management, Multidisciplinary team, Multidisciplinary telecare, Multidisciplinary treatment, tumor board, tumour board, Interdisciplinary cancer care, interdisciplinary cancer, interdisciplinary management, interdisciplinary cancer management, Multidisciplinary cancer care, Multidisciplinary cancer, Multidisciplinary management, Multidisciplinary cancer management

Search strategy used in the original (24-06-2019) and update searches (02-01-2020 and 05-01-2021).

Table S2. Search results per database.

Database	Date	Results	Total
**Pubmed**	24-06-2019	4144	4948
	02-01-2020	321	
	05-01-2021	483	
**Embase**	24-06-2019	8195	10092
	02-01-2020	890	
	05-01-2021	1007	
**Cochrane**	24-06-2019	827	1325
	02-01-2020	321	
	05-01-2021	177	
**Web of Science**	24-06-2019	3698	4547
	02-01-2020	372	
	05-01-2021	477	

Results of search strategy used in the original (24-06-2019) and update searches (02-01-2020 and 05-01-2021).

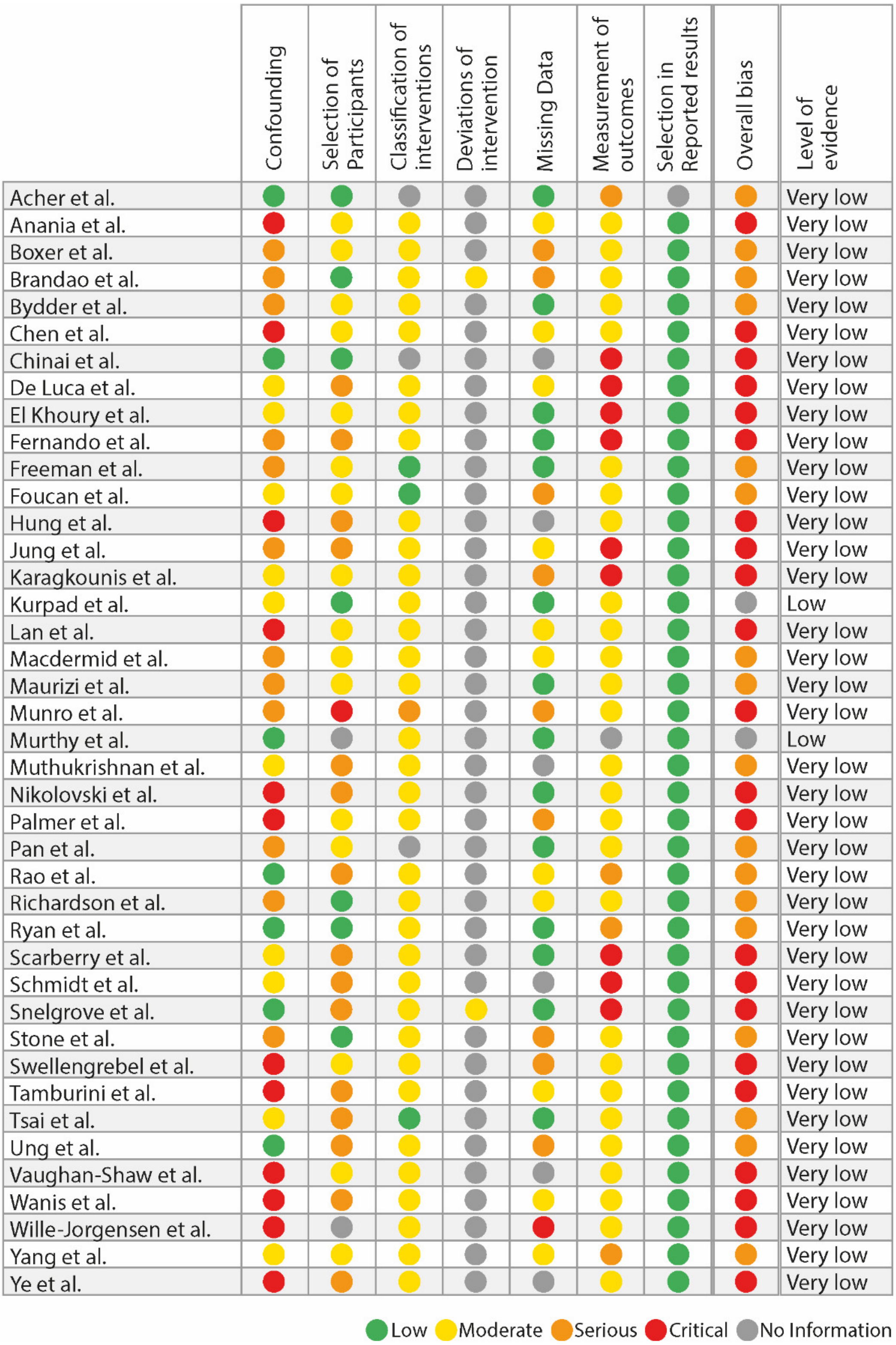

Figure S1. Risk of bias and quality of care.
