# Supplementary figures and images for: The Effects of Multidisciplinary Team Meetings on Clinical Practice for Colorectal, Lung, Prostate and Breast Cancer: A Systematic Review
Source: Cancers (Basel). 2021 Aug 18;13(16):4159. doi: 10.3390/cancers13164159 (PMC8394238; doi:10.3390/cancers13164159)

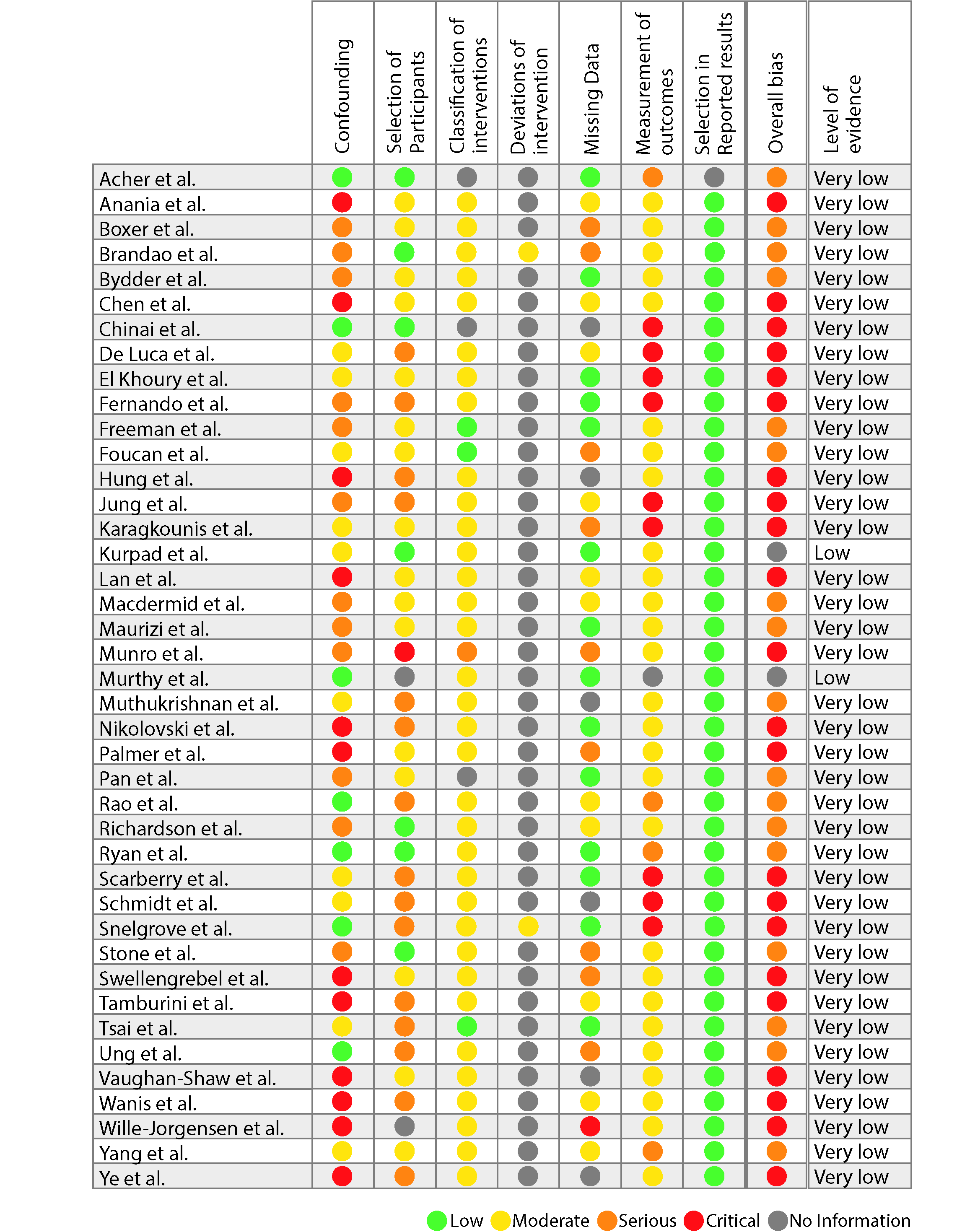

Supplement: Supplementary file 1 [file cancers-13-04159-s001.zip › Risk of Bias and level of evidencel 2020.tif]
